# Transferrin Receptor‐1: Expression in Canine Mammary Tumours and In Vitro Therapeutic Applications

**DOI:** 10.1111/vco.70000

**Published:** 2025-06-23

**Authors:** Valentina Moccia, Nicolò Rensi, Giovanni Paolo Marullo, Alessandro Sammarco, Federico Bonsembiante, Filippo Torrigiani, Lucia Salvioni, Davide Prosperi, Valentina Zappulli, Laura Cavicchioli

**Affiliations:** ^1^ Department of Comparative Biomedicine, Health and Food Science University of Padua Legnaro Italy; ^2^ Department of Animal Medicine, Production and Health University of Padua Legnaro Italy; ^3^ Department of Biotechnologies and Biosciences University of Milan Bicocca Milano Italy

**Keywords:** apoferritin nanocage, canine mammary cancer, doxorubicin

## Abstract

The transferrin receptor‐1 (TFR‐1) is overexpressed in many types of human cancers and, in recent years, several studies have investigated its use as a preferential channel for drug cellular uptake in cancer therapy. In veterinary medicine, TFR‐1 expression in cancer cells and tissues has been poorly described, as well as its therapeutic potential. In this study we investigated TFR‐1 expression in different subtypes of canine mammary tumours (CMTs) and in two CMT cell lines, one primary (CIPp) and one metastatic (CIPm). Additionally, we also compared the in vitro efficacy of an engineered human apoferritin nanocage loaded with doxorubicin (HFn(DOX)) with the conventional doxorubicin treatment on CIPp and CIPm. In CMT tissues, TFR‐1 was more expressed in the tumoral tissues compared to the hyperplastic counterparts, specifically with regards to carcinoma and malignant myoepithelioma and simple carcinoma subtypes (*p* < 0.05). CIPp and CIPm did not show any significant difference in TFR‐1 protein expression, while *TFR‐1* gene expression was higher in CIPm (*p* < 0.05). The treatment with HFn(DOX) was more efficient than free doxorubicin only on CIPp at high concentrations (50 μM). In conclusion, we show for the first time the variability of *TFR‐1* expression in CMT tissues and cell lines. Although further investigations are necessary, HFn can be loaded with different chemotherapeutic compounds, becoming an innovative therapeutic tool for the treatment of cancers highly expressing *TFR‐1* in veterinary and human medicine.

## 1 Introduction

Transferrin receptors (TFRs) are transmembrane glycoproteins that allow the internalisation of iron into cells after binding to transferrin (TF), an iron‐carrier protein [1, 2, 3]. TFR‐1 is expressed in all cells, except mature erythrocytes, and its expression increases with the cell requirement for iron [2, 3, 4]. Since iron is necessary for cell proliferation and DNA synthesis, TFR‐1 expression increases in highly proliferating cells, such as cancer cells [4]. Particularly, TFR‐1 is often overexpressed in human cancer cells, and its high expression has been associated with higher malignancy, advanced stage, poorer prognosis, and tumour recurrence in different types of human cancers [4, 5]. More than 40 years ago, TFR‐1 was demonstrated to be particularly upregulated in tissues of human breast cancer (HBC), the most common neoplasia in women [6, 7, 8]. Therefore, considering the high expression, the easy accessibility on the cell surface and the dynamics of receptor internalisation after ligand engagement, TFR‐1 has been investigated in several studies on specific anticancer therapies in human medicine [5]. In HBC, both preclinical and clinical studies have examined its therapeutic potential, aiming to overcome drug resistance and adverse toxicity of chemotherapeutics [9, 10]. Throughout the years, different drug‐delivery molecules targeting TFR‐1 have been evaluated, such as antibodies, liposomes, and nanoparticles [3]. Among these molecules, an engineered human apoferritin nanocage consisting of 24 heavy‐chain subunits (HFn) specifically exploiting TFR‐1 to internalise cells has been created [11, 12, 13] This nanocage, after loading with a compound of choice, exploits TFR‐1 to easily access the intracellular compartment of TFR‐1 expressing cells and to transfer its cargo into the cytoplasm [11].

The HFn loaded with doxorubicin (HFnDOX) has been tested in vitro, on HBC and on feline mammary tumour (FMT) cells, and in vivo in an HBC mouse model, where it has demonstrated to overcome the onset of chemoresistance, to improve the antitumor efficacy of doxorubicin and to avoid cardiotoxicity [14, 15, 16].

In veterinary medicine, preliminary studies have evaluated the expression of TFR‐1 in some types of cancers. In cats, TFR‐1 has been demonstrated to be particularly expressed in acute myeloid leukaemia immunohistochemically and in acute erythroid leukaemia by flow cytometry [17, 18]. In dogs, TFR‐1 protein expression can change according to the tumour type in lymphomas, while, in osteosarcoma, the expression increased in intratumor endothelial cells compared to the extra tumoral [19, 20]. A study performed on a heterogenous sample of dogs and cats with different tumour types showed a high but variable expression of TFR‐1 in malignant tumours by immunohistochemistry. In addition, the higher expression of TFR‐1 was correlated with a higher sensitivity to artemisinin, a phototherapeutic compound, in a canine glioblastoma cell line [21]. Differently, another study did not evidence any difference in TFR‐1 protein expression between feline and canine malignant and benign mammary tumour tissues [22]. Our research group previously explored the expression of TFR‐1 in FMTs and found the expression of the receptor was higher in metastatic tumour and metastatic lymph node tissues compared to healthy tissues. Additionally, we evaluated the therapeutic efficacy of targeting TFR‐1 in FMTs in vitro for the first time [16].

Mammary tumours are the most common neoplasia in female dogs, as HBC in women, for which they are considered a good spontaneous comparative model [23, 24, 25]. Canine mammary tumours (CMTs) are classified according to a specifically modified WHO classification recently published by the Davis‐Thompson DVM Foundation (DTF) and exhibit a wide range of morphological features leading to a variety of histopathological subtypes [24]. The primary treatment of CMTs is surgery. However, to improve the post‐operative management of aggressive CMTs, multiple therapeutic approaches have been applied but a clear consensus on adjuvant medical treatment has not been reached yet [25]. TFR‐1 expression in CMTs has been poorly investigated in relation to the tumour type and its therapeutic potential has never been evaluated. Similarly to a preliminary study of our research group on feline mammary cancer [16], here we extensively evaluated TFR‐1 expression in different subtypes of CMT tissues and in two CMT cell lines. Additionally, taking into consideration the high translational potential of HFn application in cancer therapy, we evaluate in vitro the effects of the treatment with HFn(DOX) on two CMT cell lines.

## 2 Methods

### 2.1 Histology and Immunohistochemistry

To thoroughly analyse TFR‐1 in CMTs, we compared the epithelial expression of TFR‐1 when with (complex tumours) or without (simple tumours) the association with myoepithelium, as well as in benign versus malignant lesions. Carcinoma and malignant myoepitheliomas (CMM), characterised by the proliferation of malignant myoepithelium associated with malignant epithelium, were also included [24]. Grade III carcinomas were not included because rarely diagnosed, so the study was limited to grade I and II to obtain homogeneous groups. A total of 40 canine mammary gland tissues were selected from the archive of the Diagnostic Service of Veterinary Pathology of the University of Padua, Italy. All samples were collected as part of routine clinical activity, therefore, no ethical committee approval or owner consensus were required. Samples were obtained by surgical resection and tissues were fixed in 4% formalin and embedded in paraffin (FFPE), stained with haematoxylin/eosin, and subsequently independently evaluated by two pathologists (L.C., V.Z.). Five cases for each of the following histological subtypes were selected after diagnosis according to the DTF classification [24] and grading from Peña and coauthors: [26] simple adenoma, complex adenoma, grade 1 simple carcinoma, grade 2 simple carcinoma, grade 1 complex carcinoma, grade 2 complex carcinoma, carcinoma and malignant myoepithelioma (CMM), lobular hyperplasia. In all tumour samples, surrounding lobular hyperplasia was present and evaluated in the study.

For immunohistochemistry (IHC), sections of 4 μm were prepared from all the 40 specimens to quantify and localise TFR‐1 protein expression. The staining was performed using a semiautomatic immunostainer (Ventana Benchmark XT, Roche‐Diagnostic, Basel, Switzerland). All reagents were dispensed automatically except for the primary antibody, which was added manually. A kit containing the secondary antibody and a horseradish peroxidase (HRP)‐conjugated polymer which binds mouse primary antibody (UltraView Universal DAB, Ventana Medical Systems, Oro Valley, AZ, USA) was used. An anti‐human TFR‐1 mouse monoclonal antibody (1:25, clone H68.4, #13‐6890, Thermo Fisher Scientific, Waltham, Massachusetts, USA) was used as a primary antibody and incubated for 32 min at 37°C.

The counterstaining with haematoxylin was automatically performed. A semiquantitative immunostaining evaluation was performed with an optical microscope, counting only epithelial cells in 10 random selected fields at 40× [27] and measuring membranous/cytoplasmatic immunolabelling, considering both the intensity of the staining (0 = no staining, 1 = weak staining, 2 = moderate staining and 3 = strong staining) and the percentage of positive cells. Data were then combined to obtain the H‐score, ranging from 0 to 300, according to the following formula [28]: H score = (% of negative cells × 0) + (% of weakly positive cells × 1) + (% of average positive cells × 2) + (% highly positive cells × 3).

### 2.2 Cell Culture

A primary (CIPp) and a metastatic (CIPm) canine mammary gland tumour cell lines were used [29]. Cell lines were cultured in Gibco Advanced RPMI 1640 1× (Thermo Fisher Scientific) with 10% fetal bovine serum (FBS, PAN BIOTECH, Aidenbach, Germany) and 1% penicillin–streptomycin (Corning 100 mL Penicillin–Streptomycin Solution, 100×, Corning, NY, USA). Cells were incubated at 37°C in a humidified atmosphere containing 5% CO_2_. Cells were routinely tested and confirmed to be Mycoplasma free (MycoAlert, Lonza, Basel, Switzerland).

### 2.3 Protein Extraction and Western Blotting Analysis

To evaluate the protein expression of TFR‐1 in the cell lines, a Western blotting (WB) analysis was carried out in biological triplicate. CIPp and CIPm cells were lysed with RadioImmunoPrecipitation Assay (RIPA) buffer (Thermo Fischer Scientific) supplemented with protease inhibitor (Sigma Aldrich, St. Louis, MO, USA), following manufacturer's instructions. Protein concentration was calculated with Bicinchoninc Acid Assay (Pierce BCA protein Assay Kit, Thermo Fischer Scientific) and 20 μg of proteins were used for the WB analysis. Briefly, after denaturation at 95°C for 5 min, proteins were resolved by NuPAGE 4% to 12% Bis‐Tris gel (Thermo Fisher Scientific) and transferred onto a nitrocellulose membrane (Thermo Fischer Scientific). Nonspecific binding sites were blocked by 90 min incubation at room temperature (RT) in 7% nonfat dry milk in Tris‐Buffered saline with Tween‐20 (TBS‐T; TBS + 0.1% Tween‐20). Blots were then incubated at 4°C overnight with the same antibody against TFR‐1 used for IHC (1:1000, clone H68.4, #13‐6890, Thermo Fisher Scientific), or with anti‐beta‐actin mouse monoclonal antibody (1:1000, (C4) sc‐47 778 SantaCruz Biotechnology, Dallas, TX, USA), used as housekeeping control.

After overnight incubation, the membrane was washed in TBS‐T and incubated with a peroxidase‐conjugate anti‐mouse secondary antibody (1:3000, GE Healthcare Life Science, Buckinghamshire, UK) for 1 h at RT. The reactive bands were visualised with iBright 1500 (Thermo Fischer Scientific) using a chemiluminescence detection kit (SuperSignal West Pico Chemiluminescent Substrate, Thermo Fischer Scientific). Band densities were quantified by ImageJ software (Wayne Rasband, National Institutes of Health, Bethesda, MD, USA). The relative amount of proteins was determined by normalising the densitometry value of interest to that of the housekeeping control.

### 2.4 Immunofluorescence

To confirm and localise TFR‐1 expression on CIPp and CIPm, immunofluorescence (IF) was performed. 25 000 cells were cultured on a glass coverslip in a 24‐well plate. At approximately 80% confluence, cells were washed quickly with PBS, fixed in 4% paraformaldehyde, and permeabilized with 0.1% Triton X‐100 (Sigma Chemical Co., St. Louis, MO, USA). Nonspecific binding sites were blocked with 1% bovine serum albumin (Sigma‐Aldrich, St. Louis, MO, USA). Coverslips were then incubated with the same antibody against TFR‐1 used for IHC and WB (1:250, clone H68.4 Thermo Fisher Scientific catalogue number 13‐6890) for 3 h at RT, followed by a secondary goat anti‐mouse immunoglobulin G (IgG; heavy (H) + light (L) chain) Superclonal antibody (1:2000, AlexaFluor555 conjugated, # A32727, Thermo Fisher Scientific) for 45 min at RT. Staining of nuclei was obtained with DAPI (Thermo Fisher Scientific). Images were taken with a Leica DM 4000B microscope, equipped with a Leica DC300F Camera and Leica Image Manager 50 software (Leica Microsystem, Wetzlar, Germany). TFR‐1 positivity was confirmed by the red colorimetric reaction of the cell membrane/cytoplasm.

### 2.5 Flow Cytometry

The expression of TFR‐1 in CIPp and CIPm was also analysed by flow cytometry (FC) in three biological replicates. 500 000 cells were harvested and resuspended in 500 μL of RPMI 1640 + sodium azide + FBS (1000 cells/μL). For each tube, 50 μL of cell suspension was used. Since TFR‐1 is a transmembrane receptor, cells were permeabilized using the eBioscience FoxP3/Transcription factor staining buffer set (Thermo Fisher Scientific), following the manufacturer's instructions. After permeabilization, cells were incubated for 1 h at 4°C with the anti‐TFR‐1 antibody (1:100; clone H68.4, #13‐6890, Thermo Fisher Scientific). After the incubation with the primary antibody, cell suspensions were washed and incubated with a goat anti‐mouse IgG (H + L) (1:500, Alexa flour647 secondary antibody, # A32733, Thermo Fisher Scientific) for 45 min at 4°C. Samples were washed and resuspended in 900 μL of PBS for the acquisition. To eliminate nonspecific labeling from the analyses, a separate aliquot of cell suspension, in which the secondary antibody was not included, was used as a negative control. The data acquired by flow cytometer CyFlow Space (Partec‐System, Sysmex Europe GmbH, Norderstedt‐Amburgo, Germany) were analysed with the open‐source software FCSalyzer (version 0.9.22‐alpha). For each replicate, 20 000 events were analysed. The morphology and the complexity of cells were evaluated in side scatter (SSC) versus forward scatter (FSC) scattergram, while TFR‐1‐positive cells were identified on fluorescence channel 5 versus FSC scattergram.

### 2.6 TFRC Gene Expression Through Quantitative Real Time PCR

RNA was extracted from 5 × 10^6^ CIPp or CIPm cells using the RNeasy Mini kit (Qiagen, Hilden, Germany) following the manufacturer's instructions. 500 ng of total RNA were reverse‐transcribed using the RevertAid First Strand complementary DNA (cDNA) Synthesis kit (Thermo Fisher Scientific) according to the manufacturer's instructions. The cDNA was then amplified with PowerUp SYBR Green Master Mix (Applied Biosystem, Foster City, CA; USA) and used as a template for quantitative real‐time PCR using Stratagene Mx3000 (Agilent Technologies) to evaluate the RNA expression of TFRC. Beta‐actin (ACTB) was used as a housekeeping gene. To perform the data analysis for each sample, the ΔΔCt value was calculated and expressed as a relative fold change (2^−ΔΔCt^), as described in Reference [30], considering CIPp as the reference sample. Primers were designed on an exon–exon junction using the PRIMER‐BLAST software, National Center for Biotechnology Information (NCBI) (TFRC F5 forward primer: CTTTGGACATGCTCACCTGG; TFRC F3 reverse primer: TATGCTGGGCAATCCTGACG; ACTB F5 forward primer: GATCAAGATCATCGCACCCCC; ACTB F5 reverse: GCAACTAAAGTAACAGTCCGCC).

### 2.7 Engineered HFn, FMCm Cell Treatment, and Cell Proliferation Assay

Previously characterised H‐ferritin nanocages loaded with doxorubicin (HFn(DOX)) endowed with high biocompatibility and low cellular toxicity, and an unloaded H‐ferritin nanocage (HFn) were used as the nanodrug and control, respectively [14, 15, 31, 32].

CIPp and CIPm cells were treated with different concentrations of HFn(DOX) and doxorubicin alone (DOX). The administration of HFn alone and untreated cells were used as controls. A cell proliferation assay was performed to test the effects of different treatments on cell proliferation. Briefly, 5000 cells were seeded in each well of a 96‐well plate. After 24 h, cells were treated using six drug concentrations for 24, 48 or 72 h. According to previous literature, for CIPp, concentrations of 0.01, 0.1, 1, 5, 12.08 and 50 μM were used for treatments, where 12.08 μM is described as the EC50 concentration of DOX for this cell line, while for CIPm 0.01, 0.1, 1, 5, 9.43 and 50 μM concentrations were selected, where 9.43 μM is the reported EC50 concentration of DOX [33]. After each time point, 20 μL of CellTiter 96 Aqueous One Solution cell proliferation assay (MTS, Promega, Madison, WI, USA) were added to each well and the plate was incubated for 1 h at 37°C. Absorbance was assessed at 490 nm using a spectrophotometer SpectraCount (Packard Instrument, Meriden, CT, USA).

### 2.8 Statistical Analysis

Statistical analysis was performed using GraphPad Prism 8.0 Software. Differences between two groups were tested with the two‐tailed unpaired Student's *t*‐test when data were normally distributed or the Mann–Whitney test when data were not normally distributed. To verify differences among more than two groups, one‐way ANOVA with Tukey's multiple comparison test was used when values were normally distributed. Alternatively, the Kruskal–Wallis test with Dunn's multiple comparison test was used. Normality distribution was established using a Shapiro test. The level of significance was fixed as *p* < 0.05.

## 3 Results

### 3.1 Immunohistochemistry

**FIGURE 1:**
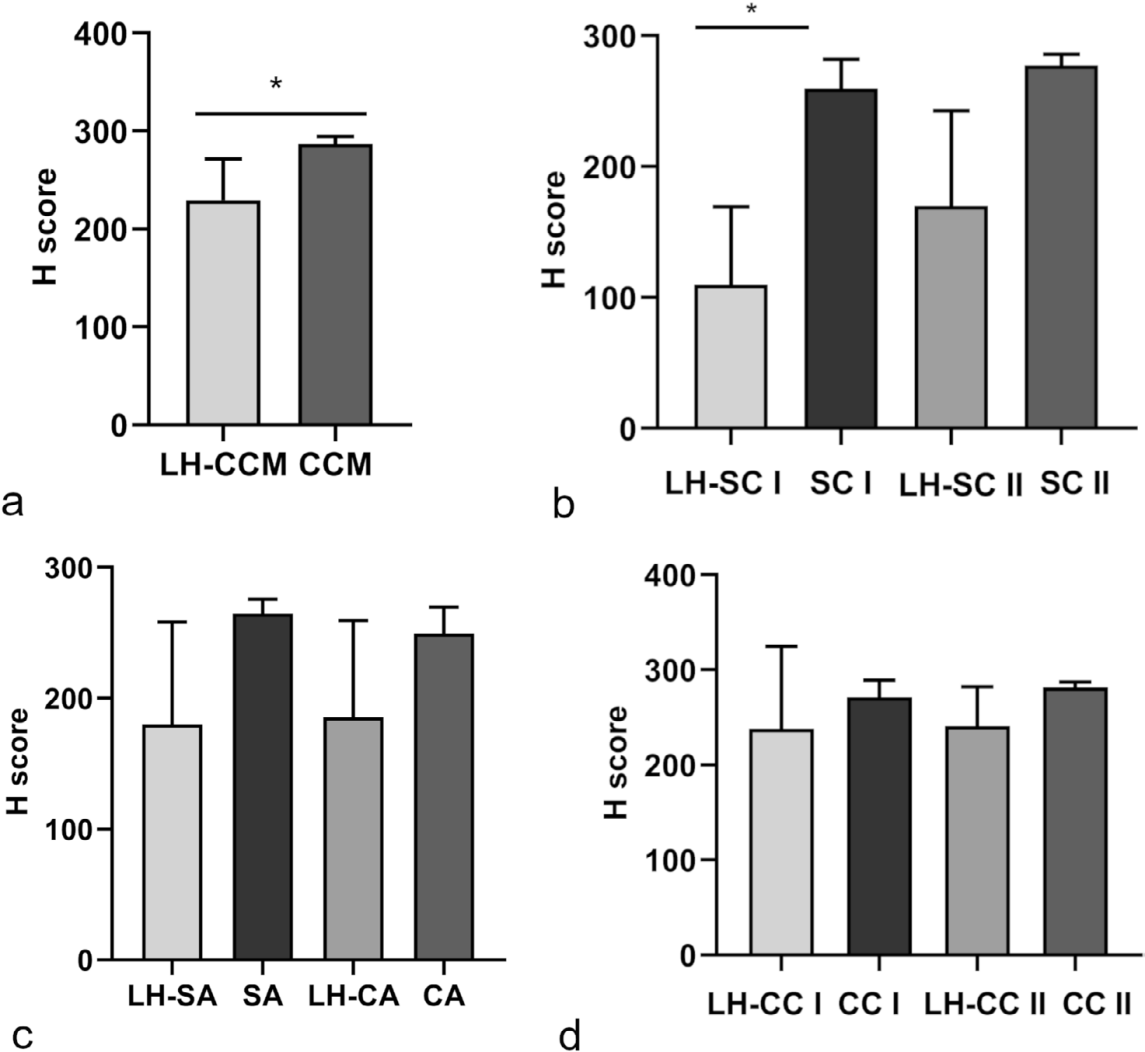
Immunohistochemical H‐score of TFR‐1 protein expression. (a) H‐score of carcinomas and malignant myoepitheliomas compared to the H‐score of the associated hyperplastic tissue. (b) H‐score of grade 1 and grade 2 simple carcinomas compared to the H‐score of the associated hyperplastic tissue. (c) H‐score of simple and complex adenomas compared to the H‐score of the associated hyperplastic tissue. (d) H‐score of grade 1 and grade 2 complex carcinomas compared to the H‐score of the associated hyperplastic tissue. CA = complex adenoma. The error bar indicates the standard deviation; CC I = grade 1 complex carcinoma; CC II = grade 2 complex carcinoma; CMM = carcinoma and malignant myoepithelioma; LH = lobular hyperplasia; SA = simple adenoma; SC I = grade 1 simple carcinoma; SC II = grade 2 simple carcinoma.

**FIGURE 2:**
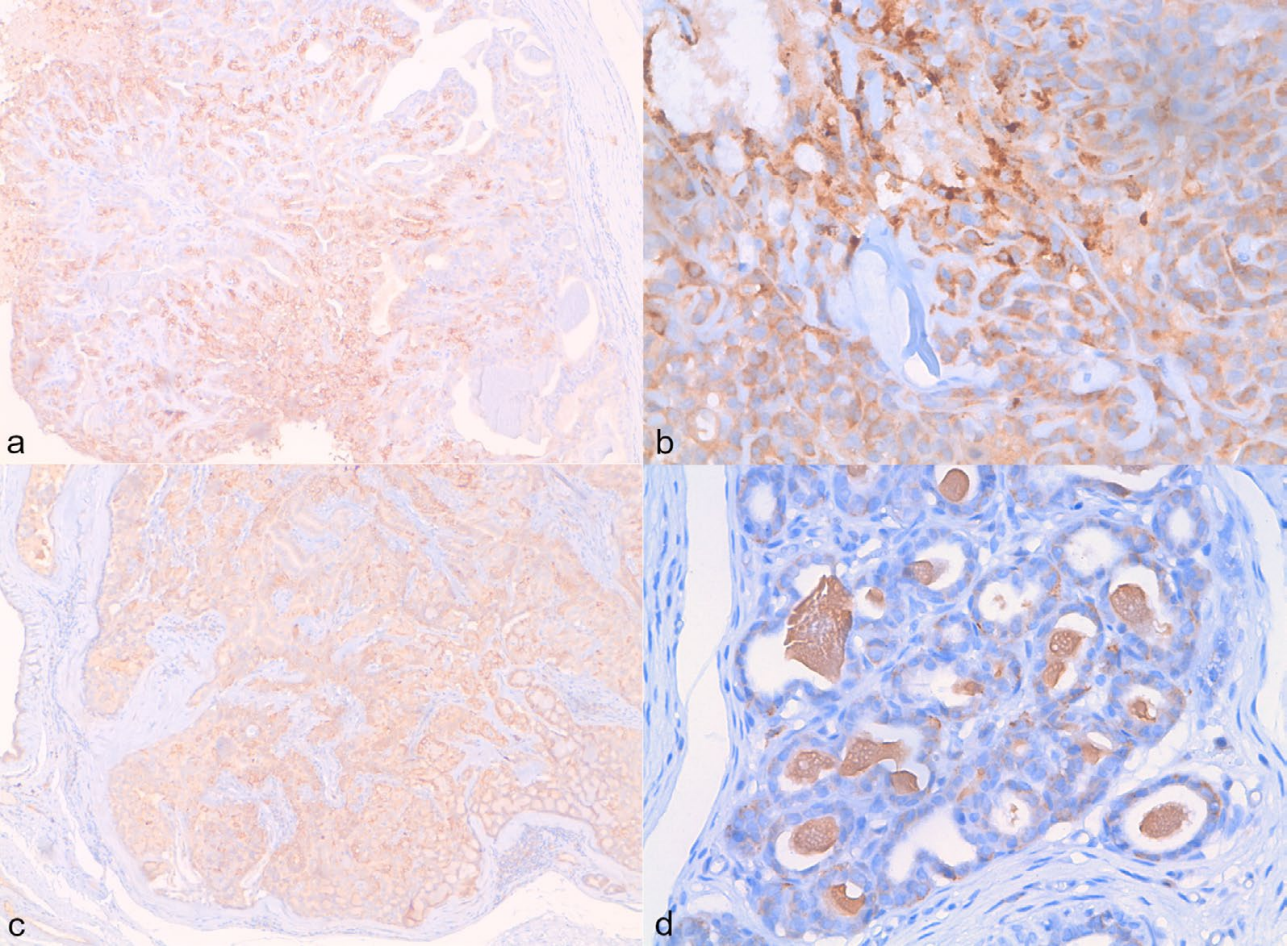
Immunohistochemistry showing TFR‐1 membrane and cytoplasmatic localization in different canine mammary lesions. (a) Simple carcinoma, 10× magnification; (b) complex carcinoma, 40× magnification; (c) Complex adenoma, 10× magnification; (d) Mammary hyperplasia, 40× magnification.

IHC analysis revealed, as expected, both cytoplasmic and membrane localization of TFR‐1 in all mammary gland tissues. The mean H‐score of each mammary gland tumour‐associated hyperplastic tissue was lower than the H‐score of the corresponding tumoral counterpart (Figure 1a–d). However, this difference was statistically significant only for grade 1 simple carcinoma, with a mean H‐score of 109.7 ± 59.3 in the hyperplastic tissue compared to a mean H‐score of 259.4 ± 31.7 in the tumoral tissue, and for CMM, where the hyperplastic tissue scored 229.3 ± 42.2 and the tumoral counterpart 287.1 ± 7.4 (Figures 1a,b and 2a–d).

### 3.2 TFR‐1 Expression in Cell Lines

**FIGURE 3:**
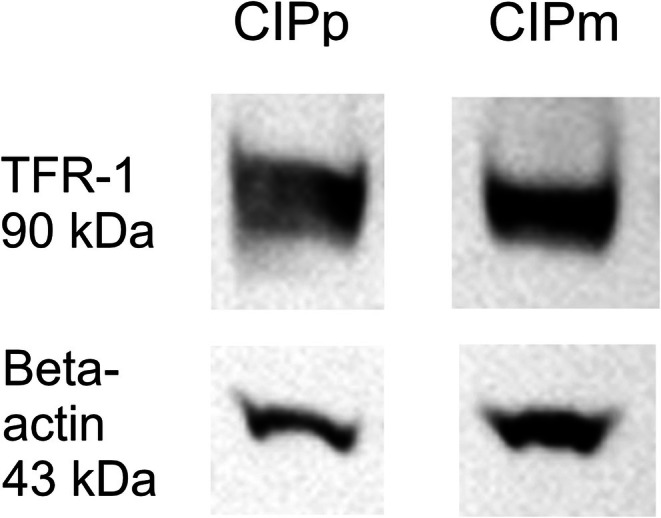
Western blotting analysis on proteins extracted from CIPp and CIPm cell lines. TFR‐1 is shown as a 90 kDa band and the housekeeping protein beta‐actin is shown as a 43 kDa band.

WB analysis showed a 90 kDa band corresponding to the expected TFR‐1 molecular weight in proteins extracted both from CIPp and CIPm (Figure 3), confirming antibody specificity. The relative quantification of TFR‐1 did not highlight any significant difference in protein expression between CIPp and CIPm.

**FIGURE 4:**
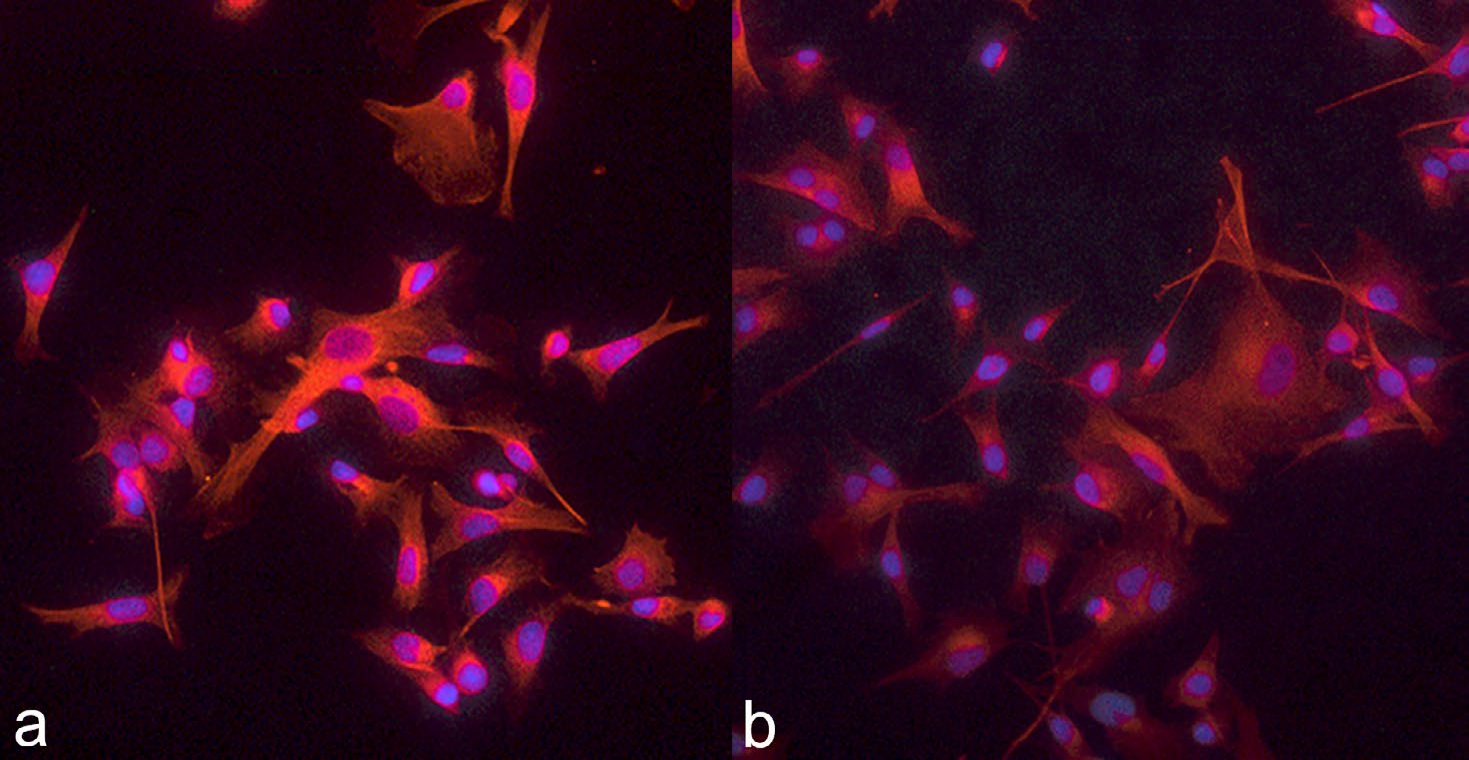
Merged images of immunofluorescence (IF) for TFR‐1 (20×). Diamidino‐2‐phenylindole (DAPI) immunostaining can be seen in blue, while TFR‐1 in red, with both cytoplasmic and membrane localization. (a) Merged images of IF carried out on CIPp; (b) Merged images of IF carried out on CIPm.

IF confirmed the expression of TFR‐1 on both CIPp and CIPm cells, with an evident fluorescence on the cell membrane and within the cytoplasm (Figure 4).

**FIGURE 5:**
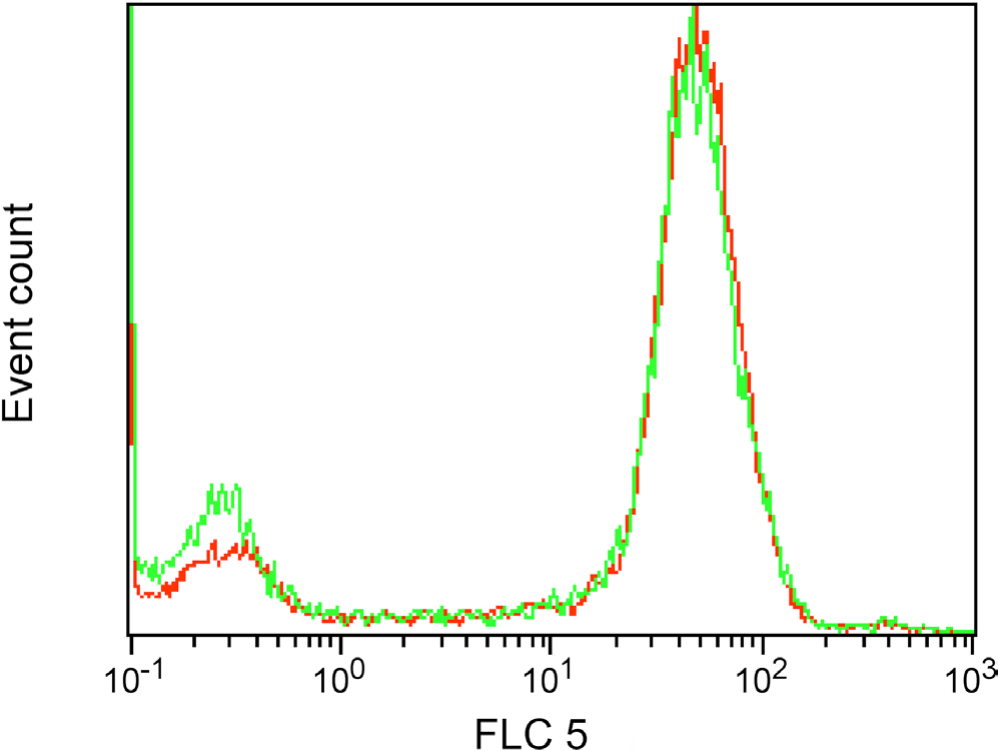
Flow cytometry for TFR‐1 performed on CIPp (green line) and CIPm (red line). No significant difference is shown in mean fluorescent intensity between the two cell lines.

At FC, Figure 5 no significant difference in the mean fluorescence intensity (MFI) of TFR‐1 between CIPp and CIPm was detected.

**FIGURE 6:**
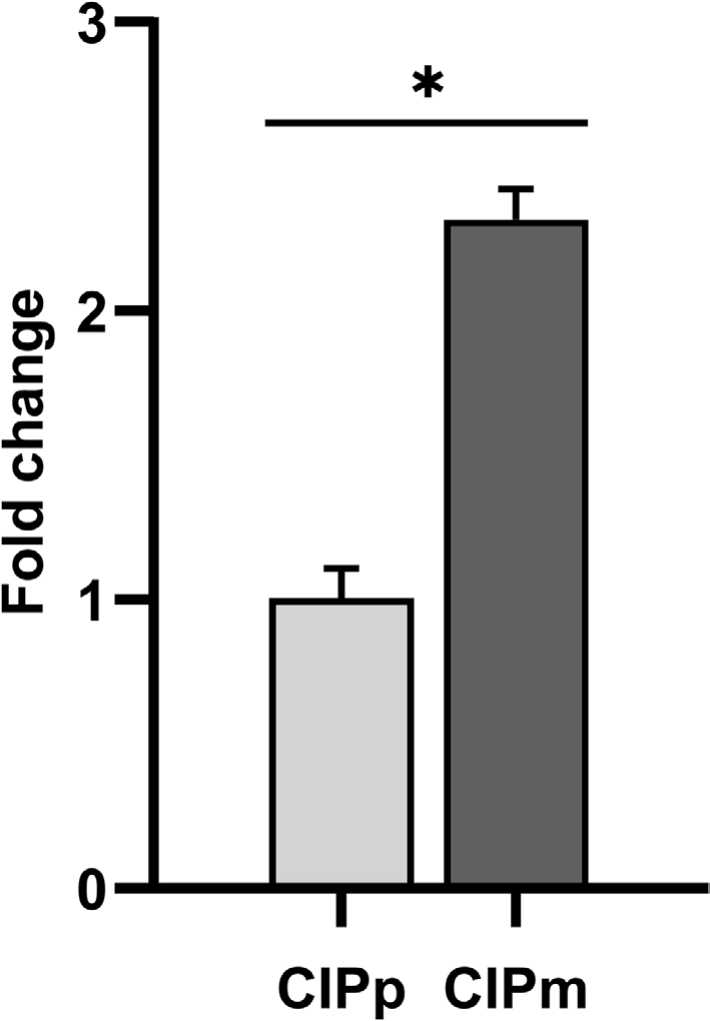
*TFRC* gene expression on CIPp and CIPm cell lines. CIPm shows a fold change value two times higher than CIPp (*p* < 0.05). The error bar indicates the standard deviation.

The evaluation of gene expression of *TFRC* by qPCR showed instead a significantly higher expression in CIPm than in CIPp (*p* < 0.05) (Figure 6).

### 3.3 Cell Proliferation Assay on Cells Treated With an Engineered HFn

To investigate the therapeutic efficacy of HFn(DOX), we performed a proliferation assay on CIPp and CIPm cell lines treated with HFn(DOX) or DOX alone.

**FIGURE 7:**
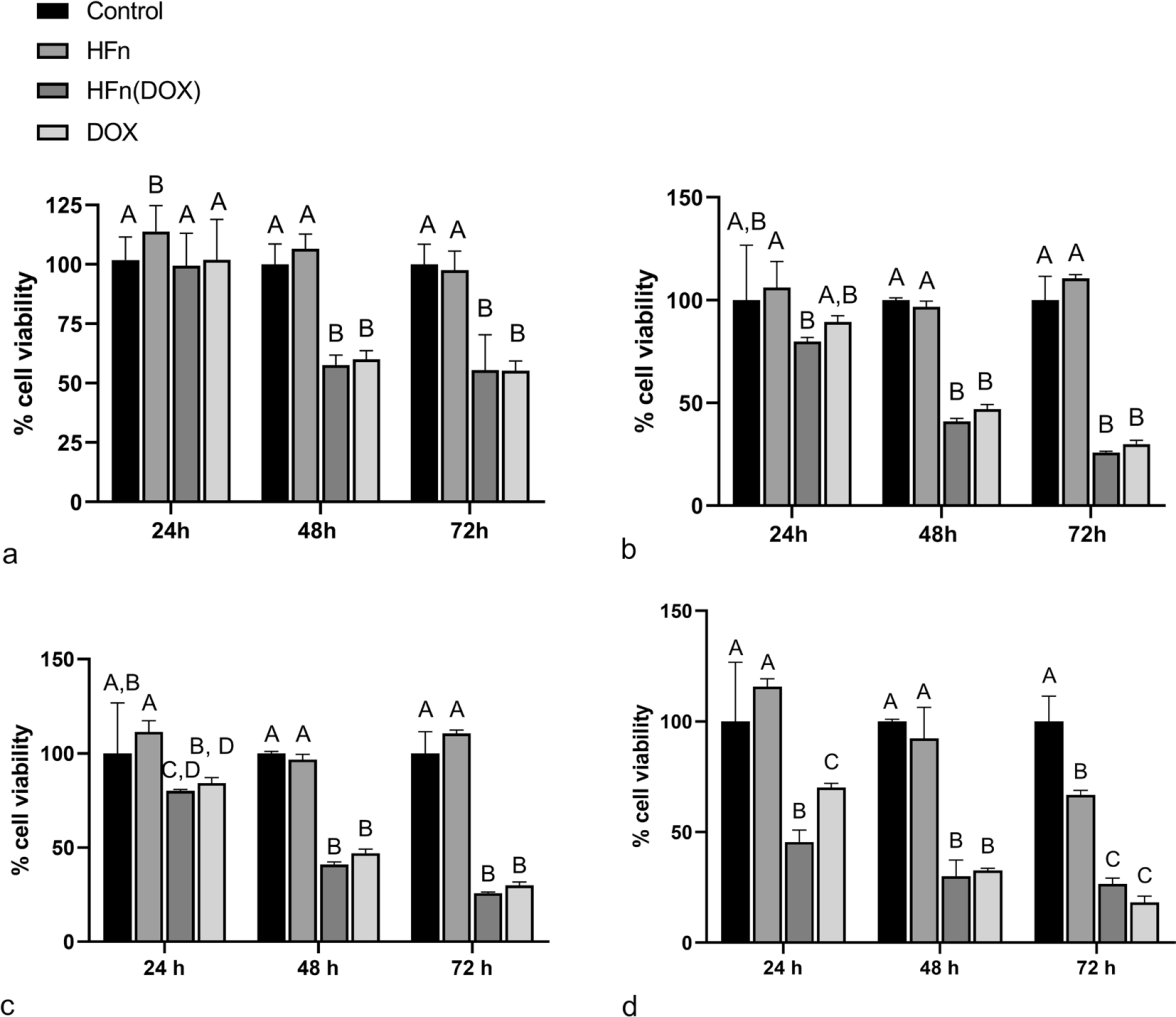
Cell proliferation of CIPp after 24, 48 and 72 h from treatment with H‐ferritin nanocages loaded with doxorubicin [HFn(DOX)], free doxorubicin (DOX), only HFn (HFn) and control at 1 μM (a), 5 μM (b), 12.08 μM (c) and 50 μM (d) concentrations. The error bar indicates the standard deviation. Groups that are not significantly different are assigned the same letter.

Both HFn(DOX) and DOX significantly reduced cell proliferation of CIPp compared to the treatment with HFn alone or control cells starting from 1 μM concentration after 48 h, an effect which was also visible at higher concentrations (5, 12.08 and 50 μM) after 48 h and 72 h of treatment (Figure 7a–d). Also, HFn alone had a slight effect on cell proliferation, which increased after 24 h of treatment at 1 μM concentration and, conversely, decreased after 72 h of treatment at 50 μM concentration (Figure 7a,d). HFn(DOX) significantly reduced cell proliferation compared to cells treated with HFn alone at 5 μM and compared to control cells at 12.08 μM concentrations after 24 h (Figure 7b,c). Treatment with HFn(DOX) significantly decreased cell proliferation of CIPp more efficiently than DOX at 50 μM concentrations after 24 h (Figure 7d).

**FIGURE 8:**
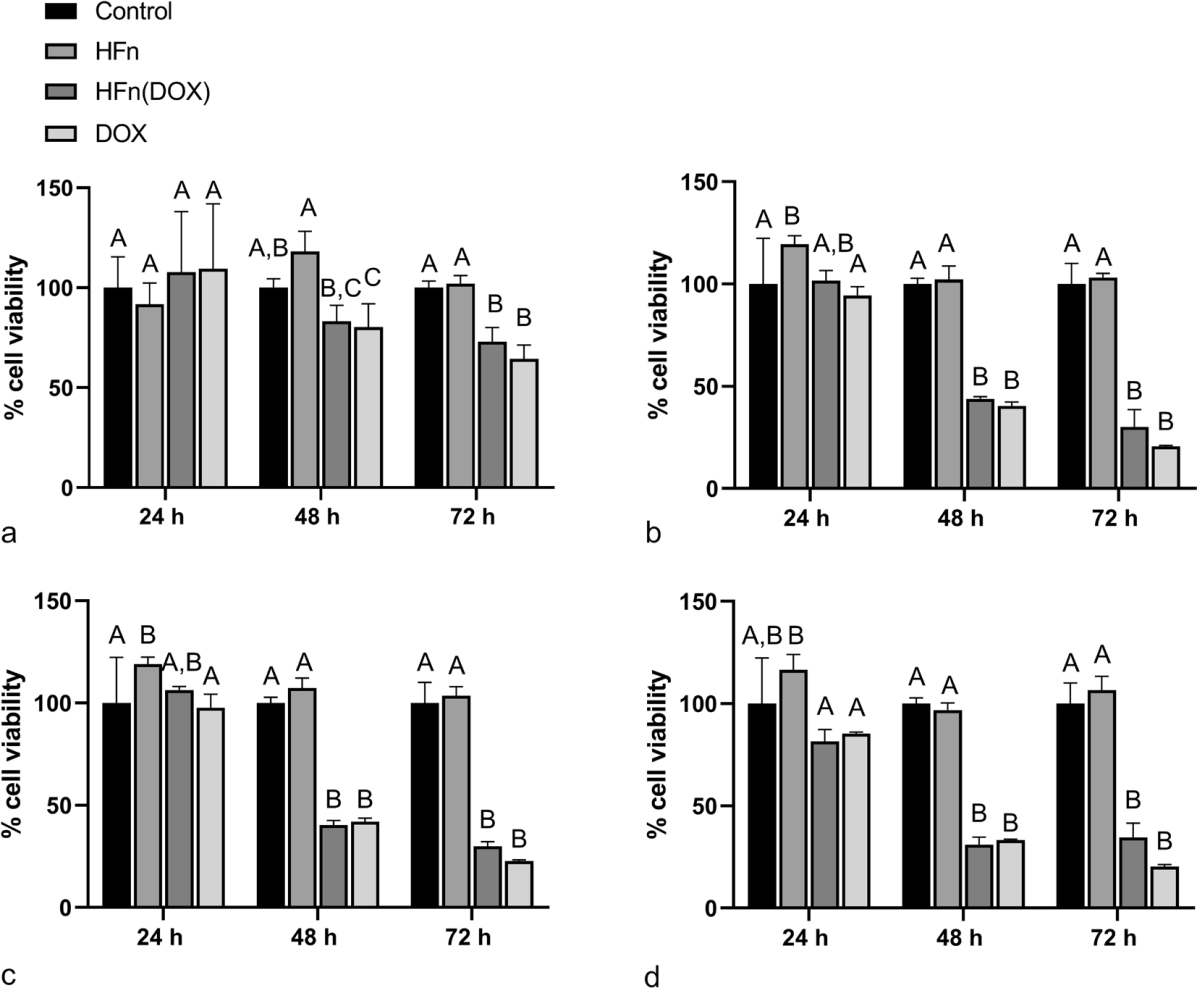
Cell proliferation of CIPm after 24, 48 and 72 h from treatment with H‐ferritin nanocages loaded with doxorubicin [HFn(DOX)], free doxorubicin (DOX), only HFn (HFn) and control at 1 μM (a), 5 μM (b), 9.43 μM (c) and 50 μM (d) concentrations. The error bar indicates the standard deviation. Groups that are not significantly different are assigned the same letter.

For CIPm, HFn(DOX) and DOX significantly reduced cell proliferation compared to untreated cells and cells treated only with HFn starting from 1 μM concentration for at least 72 h of treatment and from 5 μM concentrations for at least 48 h of treatment (Figure 8a,b). This effect was also present at higher concentrations (9.43 μM and 50 μM) for at least 48 h of treatment (Figure 8c,d). At 1 μM concentration after 48 h of treatment, both HFN(DOX) and DOX significantly reduced cell viability compared to cells treated with HFn alone, and DOX alone significantly reduced cell viability compared to control cells in the same conditions (Figure 8a). Additionally, after 24 h of treatment, DOX reduces cell proliferation compared to cells treated with HFn alone at 5 and at 9.43 μM concentrations, being more efficient than HFn(DOX) (Figure 8b,c).

## 4 Discussion

In human medicine, TFR‐1 expression in cancer and its application as an anti‐cancer therapeutic target have been extensively investigated in the last decades [3, 4, 10, 34, 35, 36, 37, 38, 39, 40].

Considering the lack of information in veterinary oncology and, particularly in CMTs, we thoroughly evaluated TFR1 expression in different CMT subtypes and, for the first time, in primary and metastatic CMT cell lines. Additionally, we also evaluated the in vitro efficacy of the treatment of two CMT cell lines with HFn(DOX), specifically targeting TFR‐1.

In this study, TFR‐1 was significantly overexpressed only in some CMT tissues in comparison to the hyperplastic counterpart (simple carcinoma and CMM). Moreover, comparing the overall expression of TFR‐1 in all tissue samples, there was only a minimal, non‐statistically significant trend of increased expression with malignancy. In animals, few studies have evaluated TFR‐1 expression in cancer tissues [16, 19, 20]. In a previous study of our research group, TFR‐1 expression increased with tumour malignancy in feline mammary carcinoma [16]. Also in women, TFR‐1 has generally been found to be overexpressed in HBC compared to healthy mammary tissues [9]. In our study, among the heterogeneity of CMTs, only simple and complex benign and malignant grade I and II tumours and CMM were included. Complex tumours are a common and peculiar canine subtype in which a benign myoepithelium proliferates together with the epithelium, resulting in a less aggressive behaviour of the malignant epithelium [24, 26]. No significant differences in the expression of TFR‐1 were noticed between simple and complex lesions or in association with malignant myoepithelium. Despite the absence of data in the literature regarding the expression of TFR‐1 in human or animal tumours with myoepithelial components, from our study it seems that the presence of mammary myoepithelium does not strongly affect the epithelial TFR‐1 expression. CMTs are well‐known to be generally less aggressive than FMTs, particularly the simple and complex subtypes [26]. Additionally, CMTs have been suggested as being a continuum from benign to malignant lesions, based on some evidences such as progressive signs of histological malignancy with increased neoplastic size in dogs with multiple tumours, the low incidence of small malignant tumours and a strong association of larger size with malignancy [41] Therefore, we might expect that some markers associated with malignancies, might progressively vary their expression during malignant transformation, showing only a slighter increase in the initial phases and in less aggressive cases (i.e., grade I/II tumours and less aggressive phenotypes). All these aspects might explain the minimal increase of TFR‐1 in association with malignancy in this study compared to FMTs. Therefore, grade III CMTs should also be investigated in additional studies. Moreover, in previous studies on HBC, TFR‐1 has manifested a highly variable expression in relation to the tumour subtype, to patient characteristics, and within the cells of the same tumour [9]. Considering the high heterogeneity of CMTs, other CMTs subtypes that can be very aggressive (i.e., solid and anaplastic carcinoma) should be further investigated for TFR‐1 expression [24].

In CMT cell lines, TFR‐1 presence was demonstrated by WB, IF, and FC in both CIPp and CIPm. qRT‐PCR showed that TFR‐1 expression was greater in CIPm than in CIPp. While CIPp originates from a primary mammary tumour, CIPm derives from the corresponding lymph node metastasis [29]. The higher expression of TFR‐1 at qRT‐PCR in the metastatic cell line is coherent with the human literature, where TFR‐1 has been found to be upregulated in different metastatic tumour tissues, including HBC, further suggesting a relevant role of TFR‐1 targeting therapies [5, 39].

Considering the evaluation of the efficacy of HFn(DOX) treatment in vitro, both HFn(DOX) and free DOX caused a reduction in cell proliferation starting from treatment concentrations of 1 μM after 48 h of incubation in CIPp and after 72 h in CIPm. The need for a high concentration of DOX, also when using the HFn, to reduce cell proliferation means a general low efficacy of DOX, probably related to the onset of a multidrug resistance phenotype which has already been demonstrated in these cell lines [33].

Additionally, in our study a significant different effect on cell viability between HFn(DOX) and DOX was evident only in the CIPp cell line at high concentrations (50 μM) after 24 h, where HFn(DOX) reduced cell proliferation more efficiently than DOX. Additionally, in the CIPp cell line, HFn(DOX) was more efficient than DOX in reducing cell viability compared to control conditions after 24 h of treatment at 5 and 12.08 μM concentration. Conversely, in the CIPm cell line, was DOX the more efficient treatment to reduce cell proliferation compared to control conditions, specifically after 24 h of treatment at 5 and 9.43 μM concentrations. Therefore, HFn(DOX) seems to have a higher efficacy only on the CIPp cell line, specifically after short incubations (24 h). This is partially in contrast with qRT‐PCR results, showing a higher expression of TFR‐1 in CIPm. In our study, the MFI quantification at FC and the relative quantification at WB did not highlight significant difference in TFR‐1 protein expression between the two cell lines. Therefore, the high gene expression levels measured in CIPm by qRT‐PCR probably do not correspond to a higher protein expression than in CIPp. However, to achieve a potentially successful treatment, TFR‐1 absolute protein quantity should be determined.

In a preliminary study of our research group performed on a FMT cell line, the antiproliferative effect of HFn(DOX) was already visible at 0.01 μM whereas DOX effects started from concentrations of 0.1 μM, showing a higher sensitivity of the cell lines to DOX and a higher efficacy of HFn(DOX) compared to our study [16]. For the treatment of FMTs, clinical data on the efficacy of DOX as adjuvant therapy are few and report contrasting outcomes [42, 43], while for CMTs, DOX is generally not considered an appropriate treatment when not used in association with other chemotherapeutics for the arising of chemoresistance [44, 45]. Nevertheless, the application of HFn to reduce neoplastic cell growth and side effects of chemotherapy simultaneously, should not be completely discouraged.

As an example, some studies performed on HBC cell lines have demonstrated the pro‐apoptotic effect against cancer cells of apoferritin loaded with other chemotherapeutic agents, such as the natural molecule curcumin, an antineoplastic, anti‐inflammatory and chemosensitizer compound [36, 46, 47]. The latter effect has been demonstrated after combination of curcumin with DOX, resulting in a reduced efflux of DOX in DOX‐resistant HBC cell lines [48]. Interestingly, two studies have shown that curcumin loaded in natural horse‐derived apoferritin or in synthetic apoferritin nanoparticles combined with quercetin was efficiently delivered into MCF‐7 cells, with consecutive strong antiproliferative effects [46, 47].

To better understand the efficacy of the application of HFn in veterinary oncology, further studies using different chemotherapeutic compounds on different tumour subtypes should be performed.

## 5 Conclusions

In the present study we demonstrated for the first time the variability of TFR‐1 expression in a subset of canine mammary gland tissues and in two CMT cell lines, presenting also the in vitro treatment of CMT cells with a HFn(DOX). HFn loaded with an effective chemotherapeutic, can be an innovative therapeutic tool specifically targeting cancer cells highly expressing TFR‐1 in different animal species and cancer subtypes.

## Author Contributions

Conceptualization: V.Z., L.C., and D.P. Methodology: V.M., N.R., A.S., F.B., L.S., D.P., V.Z., and L.C. Data curation: N.R., V.M., and G.P.M. Formal analysis: V.M. and N.R. Writing – original draft: V.M. Writing – review and editing: V.M., N.R., G.P.M., A.S., F.B., F.T., L.S., D.P., V.Z., and L.C. Supervision: L.C. Project administration: L.C. and V.Z.

## Conflicts of Interest

The authors declare no conflicts of interest.

## Data Availability

The data that support the findings of this study are available from the corresponding author upon reasonable request.
